# Antidepressant Effect of Aminophylline After Ethanol Exposure

**DOI:** 10.3797/scipharm.1208-17

**Published:** 2012-10-23

**Authors:** Sarah Souza Escudeiro, Paula Matias Soares, Anália Barbosa Almeida, Rodrigo de Freitas Guimarães Lobato, Dayane Pessoa de Araujo, Danielle Silveira Macedo, Francisca Cléa Florenço Sousa, Manoel Cláudio Azevedo Patrocínio, Silvânia Maria Mendes Vasconcelos

**Affiliations:** 1 Departament of Physiology and Pharmacology, Federal University of Ceará, Rua Cel. Nunes de Melo 1127, CEP 60431-270, Fortaleza, Ceará, Brazil.; 2 Superior Institute of Biomedical Sciences, Academic Master in Physiological Sciences, State University of Ceará, Av. Paranjana 1700, CEP 60740-000, Campus do Itaperi, Fortaleza, Ceará, Brazil.; 3 Christus Medicine Faculty, Rua Israel Bezerra 630, CEP 60135-460, Fortaleza, Ceará, Brazil.

**Keywords:** Adenosine, Ethanol, Aminophylline, Monoamines, Behavior, Prefrontal cortex

## Abstract

This work investigated the association of acute ethanol and aminophylline administration on behavioral models of depression and prefrontal monoamine levels (i.e. norepinephrine and dopamine) in mice. The animals received a single dose of ethanol (2 g/kg) or aminophylline (5 or 10 mg/kg) alone or in association. Thirty minutes after the last drug administration, the animals were assessed in behavioral models by the forced swimming and tail suspension tests. After these tests, the animals were sacrificed and the prefrontal cortices dissected to measure monoamine content. Results showed that ethanol presented depression-like activity in the forced swimming and tail suspension tests. These effects were reversed by the association with aminophylline in all tests. Norepinephrine and dopamine levels decreased, while an increase in the dopamine metabolite, (4-hydroxy-3-methoxyphenyl)acetic acid (DOPAC), after ethanol administration was observed. On the contrary, the association of ethanol and aminophylline increased the norepinephrine and dopamine content, while it decreased DOPAC when compared to the ethanol group, confirming the alterations observed in the behavioral tests. These data reinforce the involvement of the adenosinergic system on ethanol effects, highlighting the importance of the norepinephrine and dopamine pathways in the prefrontal cortex to the effects of ethanol.

## Introduction

Ethanol differentially interferes with the transmission processes in the central nervous system, affecting neurotransmitters [1, 2] and leading to a variety of behavioral and physiological changes such as motor incoordination, memory impairment, anxiety reduction, and depression, among others [3–5]. A significant proportion of alcohol-dependent individuals suffer from affective disorders such as depression [6–10]. However, the onset of major depression after alcohol dependence/abuse does not necessarily imply a causative relationship, and the pathogenesis remains obscure.

Among the wide range of pathways in the central nervous system that are modified by ethanol, it is important to highlight those that underlie ethanol’s diverse effects, like the ones related to the release of gamma-amynobutiric acid (GABA), glutamate, dopamine, and norepinephrine [11, 12]. Moreover, another pathway that is of increasing interest in research about ethanol’s effects is the adenosine system [13–15].

Adenosine was described as a potent depressor of neuronal activity [16], and acts mainly via the A_1_ receptor, which is a presynaptic inhibitor of the release of neurotransmitters such as dopamine, GABA, glutamate, acetylcholine, and norepinephrine [17–19]. Moreover, adenosine is involved in behavioral processes such as motor function, anxiety, depression, reward, and drug addiction, as well as in human disorders, for instance, Parkinson’s disease and schizophrenia [20]. Indeed, adenosine plays an important role in affective disorders. Clinical and experimental evidence showed that reduced adenosinergic activity is involved in bipolar mania and aggressive behavior [21] and A_2A_ receptors have been implicated in panic disorders [22].

In addition, there is strong evidence for the involvement of the adenosinergic system on ethanol effects, including: i) the increase in extracellular adenosine levels after acute ethanol exposure [23, 24], ii) the potentiation or blockade of ethanol-induced motor incoordination provided by adenosine receptor agonists or antagonists, respectively [5, 25], and iii) the reduction of anxiogenic-like behavior after acute ethanol withdrawal [13]. Adenosine antagonists, like caffeine, are implicated in alcohol tolerance [26] and in the retrograde memory impairment caused by ethanol [27]. Thus, adenosine receptors seem to modulate some of the pharmacological properties of ethanol, interacting with this drug with a resulting blockade or potentiation of its properties.

The adenosine antagonist aminophylline is an established drug in the clinical treatment of asthma and consists of approximately 80% of theophylline, its major active compound, which shows a central excitatory effect [28]. Another clinical use of aminophylline, due to its stimulatory effect, occurs in anesthesia to accelerate the consciousness recovery process after general anesthesia [29].

Because ethanol causes depression-like behavior, the present study investigated the ability of aminophylline, a non-selective adenosine receptor antagonist, to reverse ethanol’s behavioral alteration using the tail suspension and forced swimming tests. Monoamine levels (i.e. norepinephrine and dopamine) in mice prefrontal cortices were also evaluated.

## Materials and methods

### Animals and drugs

Male Swiss mice (n= 6–14) weighing 25–30 g from the Animal House of the Federal University of Ceará were used. The animals had free access to a commercial diet (Purina, Brazil) and water, and were housed in groups of 10 in a room with a 12 h on-and-off lighting schedule. Experiments were performed according to the Guide for the Care and Use of Laboratory Animals, from the US Department of Health and Human Services, and approved by the Ethics Committee for Animal Use of the State University of Ceará (protocol nº 08476336-1).

A 20 percent ethanol (Merck, Germany) solution (w/v) was administered orally (p.o.), with an intragastric cannula, at 2 g/kg body weight. Aminophylline (Brazilian Teuto Laboratory S/A, Brazil) was administered intraperitoneally (i.p.), at 5 or 10 mg/kg body weight. All drugs were diluted in distilled water.

### Experimental procedure

Mice were treated with distilled water (controls), ethanol (E: 2 g/kg, p.o.), or aminophylline (A: 5 or 10 mg/kg, i.p.). For the association protocol (E/A), mice were pre-treated with ethanol 30 minutes before aminophylline (5 or 10 mg/kg, i.p.) administration. All drug doses, as well as the sequence of administration, were chosen according to previous studies in our laboratory [5, 30].

The effects of ethanol and aminophylline alone or in association were studied in mice behavioral models (forced swimming and tail suspension tests) 30 min after the last drug administration, and the monoamine concentrations were determined after the tests. Different groups of animals were used for each test.

#### Forced swimming test

To evaluate the antidepressant activity of the treatment with ethanol and/or aminophylline, the Porsolt protocol [31] was used which includes two exposures to a water tank, spaced 24 hours apart. For these experiments, the tank size was 22 cm in diameter and 40 cm in height. The tank had a rounded lid and contained 20 cm high fresh water at 25 °C. During the first exposure, mice were placed in the tank and left there for 15 min. During the second exposure (test session), mice were placed in the tank and left there for 5 min during which immobility time was registered. A mouse was considered immobile when it remained floating in the water, without struggling, making only very slight movements necessary to keep its head above water. Each animal was tested once.

#### Tail suspension test

The tail suspension test is a screening procedure to detect antidepressant activity of drugs in rodents. The total duration of the test (6 min), as originally proposed by Steru, Chermat [32], can be divided into periods of agitation and immobility. Antidepressant drugs decrease the duration of immobility time, as do psychostimulants. In the present protocol, mice were suspended on the edge of a shelf, 58 cm above a table top, by an adhesive tape placed approximately 1 cm from the tip of their tails. The duration of immobility was recorded for a 6 min period.

#### Determination of monoamine concentrations

For determination of monoamine concentrations, the groups were sacrificed after the tests, and the prefrontal cortex was dissected on ice for the preparation of 10% homogenates (10% w/v) that were sonicated in 0.1 M HClO4, for 30 sec and centrifuged at 4 °C for 20 min at 14000 RPM. A 20 μL sample of the supernatant was then analyzed by high performance liquid chromatography (HPLC). The mobile phase was 0.163 M citric acid (pH 3.0) containing 0.02 mM EDTA, with 0.69 mM sodium octanesulfonic acid (SOS), as an ion pairing reagent, 4% v/v acetonitrile, and 1.7% v/v tetrahydrofuran.

Prefrontal cortex concentrations of norepinephrine (NE), dopamine (DA), and its metabolite (4-hydroxy-3-methoxyphenyl)acetic acid (DOPAC), were detected electrochemically using an amperometric detector (Shimadzu, Japan) by oxidation on a glassy carbon electrode at 0.85 V relative to the Ag–AgCl reference electrode and results were expressed as ng/g wet tissue.

### Statistical analyses

In the present study, all results are presented as the mean ± standard error media (S.E.M). Data were analyzed by One-Way ANOVA followed by Tukey as a *post hoc* test. Results were considered significant at p < 0.05. All tests were performed using the GraphPad Prism 5.0 software package.

## Results

### Forced swimming test

**Fig. 1:**
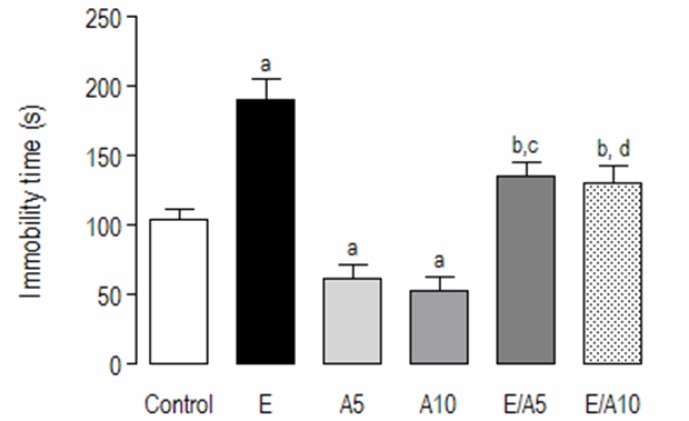
Evaluation of the potential antidepressant-like activity of the association of ethanol and aminophylline using the forced swimming test. Animals were preconditioned 24h before the test, being exposed for 15 min in a water tank, without drugs. On the test day, 30 minutes after the drug administration, animals were tested in the forced swimming test, with the immobility time as the parameter observed over 5 minutes. a, b, c, d mean statistically significant differences, as related to the Control, E, A5, and A10 groups, respectively. p<0.05 (ANOVA followed by Tukey as the *post hoc* test). (Abbreviations: E: ethanol, A: aminophylline).

Figure 1 shows an increase in the immobility time in E-treated animals, while aminophylline in both doses studied decreased this parameter as compared to the controls (C: 104.8 ± 6.9; E: 190.8 ± 14.3; A5: 62.3 ± 9.4; A10: 53.7 ± 8.9). In the groups that received the association (E/A), the immobility time was reduced (E/A5: 135.6 ± 10.2; E/A10: 130.4 ± 12.4) as compared to the ethanol group, but increased when compared with aminophylline alone [F (5,69) = 23,84; p<0.0001].

### Tail suspension test

**Fig. 2:**
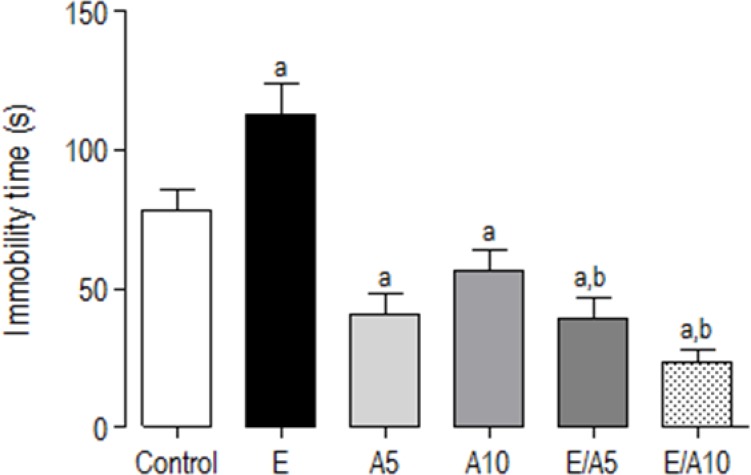
Evaluation of the potential antidepressant activity of the association of ethanol and aminophylline using the tail suspension test. Thirty minutes after the drug administration, the animals were tested in the tail suspension test, with the immobility time as the parameter observed over 6 minutes. a, b mean statistically significant differences, as related to the Control and E groups, respectively. p<0.05 (ANOVA followed by Tukey as the *post hoc* test). (Abbreviations: E: ethanol, A: aminophylline).

In the tail suspension test, an increase in immobility time was observed in the E group, while aminophylline (5 and 10 mg/kg) decreased this parameter when compared to the controls (C: 78.5 ± 7.6; E: 112.8 ± 11.6; A5: 40.7 ± 7.4; A10: 56.9 ± 7.1). Furthermore, both association groups presented a reversal of ethanol’s depression-like effect, thus showing a decrease in the immobility time as compared to E and the control groups (E/A5: 39.4 ± 7.4; E/A10: 23.5 ± 4.4) [F (5,79) = 13,51; p<0.0001] (Figure 2).

### Monoamine levels

**Fig. 3:**
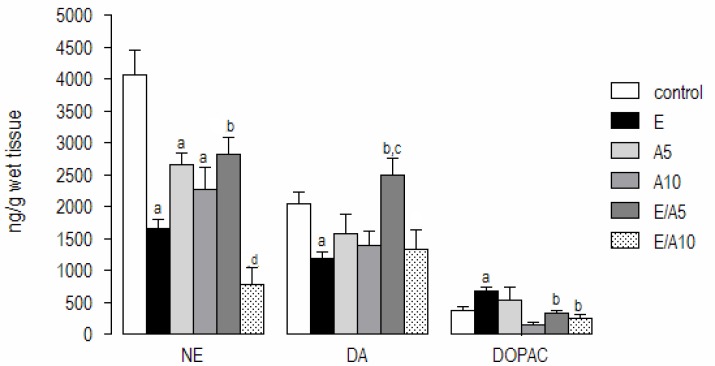
Effects of ethanol associated with aminophylline on monoamine levels in mice prefrontal cortices. Data are presented as the mean ± S.E.M. (n = 4–14). a, b, c, d, and e mean statistically significant differences with p < 0.05 as compared to the controls, A5, A10, and E/A5 groups, respectively. NE, norepinephrine; DA, dopamine; DOPAC, 4-hydroxy-3-methoxy-phenylacetic acid.

The levels of NE, DA, and DOPAC in the prefrontal cortex are presented in Figure 3. The results showed that NE and DA content decreased in ethanol-treated groups as compared to the control group [F (5,64) = 14,85; p<0.0001]. The decrease in NE and DA content seen in the ethanol group was reversed by the association with aminophylline only in the lower dose (E/A5: 2820 ± 266.9), which increased the levels of these monoamines by 70% (NE) and 97% (DA) when compared to the ethanol group (NE: 1657 ± 131.1; DA: 1180 ± 103.8). Aminophylline alone, in both doses, did not alter the levels of these monoamines.

Analyzing the monoamines, metabolites, and ethanol (677.6 ± 59.1) increased the DOPAC levels as compared to the control (C: 362.8 ± 67.2) and the association groups (E/A5 or E/A10). This drug was also effective in the reversal of the metabolite alterations caused by ethanol, reducing the DOPAC concentration when compared to the ethanol group (E: 677.6 ± 59.1; E/A5: 322.6 ± 39.9; E/A10: 241.0 ± 63.2) [F (5,54) = 5,42; p<0.0005].

## Discussion

We investigated the effects of the association of acute ethanol and aminophylline administration in behavioral models for the assessment of depression-like activity, i.e. forced swimming and tail suspension tests, which are predictive models of clinical antidepressant activity. Because regulations in monoamine levels are related to depression-like activity, we also assessed monoamine levels in the prefrontal cortex.

In the present work, the administration of ethanol in mice induced a depression-like behavior, as indicated by a significant increase in the immobility time in the forced swimming and tail suspension tests. The comorbidity of substance use disorders (for example ethanol) and depression is highly prevalent in the general population [7, 33, 34]. On the other hand, aminophylline alone demonstrated an antidepressant-like action, thus presenting an opposite effect when compared to ethanol alone in almost all behavioral parameters analyzed. Worthy of mention, is our results have demonstrated that aminophylline is able to reverse ethanol depression-like behavior.

Aminophylline is a non-selective adenosine receptor antagonist [35, 36]. There is evidence that adenosine is a neuromodulator which takes part in a variety of processes in both physiological and pathological conditions. Adenosine and its analogues tend to produce depression-like behavior in animal models [37]. Indeed, adenosine and 2-chloroadenosine increased the immobility time of mice subjected to the forced swim test [38], while classical antidepressants have been found to reverse adenosine-mediated immobility [39]. It was shown that adenosine A_2A_ receptor knockout mice displayed reduced immobility in functional assays *in vivo*, such as in the tail suspension and forced swim tests [37].

In addition, an increasing body of evidence points to a direct role of adenosine in mediating some of the cellular and behavioral responses to ethanol [15, 40]. Caffeine and selective adenosine receptor antagonists reduce the duration of ethanol-induced loss of the righting reflex [38, 41], block the motor incoordination promoted by ethanol [5, 25, 42], and reverse retrograde memory impairment caused by a high dose of ethanol (3 g/kg) [27]. Indeed, adenosine A1 receptors modulate the anxiolytic-like actions of ethanol [13], and it has been suggested that the reinforcing properties of ethanol are in part mediated via A2 activation of cyclic adenosine monophosphate/phosphokinase A signaling in the nucleus accumbens, predicting that the administration of the A2a antagonist might reduce ethanol reward and consumption [15].

Considering the alterations in monoamine levels, the results showed that the depression-like behavior produced by ethanol in the behavioral tests was followed by a decrease in norepinephrine and dopamine content in the prefrontal cortex, and this effect was reversed by aminophylline treatment, only in the lower dose.

According to the monoamine theory of depression, depressive disorders could be a result of low concentrations of monoamines such as norepinephrine, dopamine, and serotonin in putative brain areas [43, 45]. The depression-like behavior observed in our results after ethanol administration could be a consequence of a decrease in monoamine levels. However, it is noteworthy that ethanol’s action in the brain is complex with a wide range of neurotransmitters involved in what can lead to a variety of behavioral and physiological alterations like sedation, amnesia, motor incoordination, depression mood, seizure, and others [3, 5, 46].

Adenosine is an important neuromodulator of the CNS. In fact, this purine nucleoside can modulate a variety of neurotransmitters such as norepinephrine, dopamine, and serotonin [19]. The modulation of the dopaminergic system occurs through its interaction with dopaminergic receptors, and it is thought that this antagonistic interaction between adenosine A_2A_/dopamine D2 and adenosine A_1_/dopamine D1 receptors is at least partly responsible for the motor stimulant effects of adenosine receptor antagonists such as caffeine, and for the motor depressant actions of adenosine receptor agonists [47]. Dopaminergic neurons projecting to the prefrontal cortex provide direct and indirect inhibition of excitatory output to subcortical regions thought to be involved in the initiation of motor activity. Thus, decreased dopamine transmission would enhance, while increased transmission would attenuate the response to psychostimulants [48]. According to this evidence, from our results we could observe that ethanol decreased dopamine levels, and this effect was reversed by the association with aminophylline only in the lower dose, also suggesting that aminophylline’s effect could be dose-related.

Regarding the metabolites analyzed, we observed that DOPAC levels, the main dopamine metabolite in rodents, were increased after ethanol administration. A similar finding was presented by Myung et al. [4] in a study that evaluated the memory-enhancing abilities in ethanol-treated animals, in which the levels of some neurotransmitters were significantly changed by ethanol. This data suggests that there was an increase in dopamine’s metabolic rate induced by ethanol. However, in the association group, DOPAC levels returned to the control ones, thus showing that aminophylline treatment is able to reverse the increase in dopamine metabolic rate induced by ethanol.

## Conclusion

Ethanol produced a depression-like behavior as well as decreased the levels of NE and DA in the prefrontal cortex of mice, and these effects were reversed by the administration of aminophylline, a non-selective adenosine receptor antagonist.
